# Diseases of the Fingers: C.—Contractions

**Published:** 1891-08-15

**Authors:** 


### SURGERY.

### DISEASES OF THE FINGERS.

C.—Contractions.

These are very common, and may be conveniently classified according to the tissue or part primarily affected, thus:—(1) Contractions of fascia or connective tissue, including the kind described by Dupuytren and called after him; (2) contractions of the skin; (3) of the tendons; (4) of the muscles (including paralytic and spastic conditions; (5) of the ligaments between the metacarpal bones and phalanges or of the inter-phalangeal joints; (6) malformations in the bones themselves; and (7) conditions due to nervous disease.

1. Besides the so-called palmar fascia, which radiates towards the webs of the fingers from its origin at the insertion of the Palmaris longus, there is a fairly strong covering over the fingers themselves called Gerdy's fascia. These constantly participate in various injuries to the hands; and inflammation in either is apt to lead to contraction unless this is obviated by the use of splints. Burns and scalds by boiling oil, &c., are consequently frequent causes of extensive deformity. Punctured wounds which are followed by the formation of pus and constant irritation of the parts by the use of certain tools are also apt to cause contractions. In all these traumatic conditions the possibility of this result should be borne in mind; and the use of splints at first and of friction and passive movement afterwards should be adopted.

All fascial contractions due to traumatic causes are sometimes spoken of as "false Dupuytren's disease" in contradistinction to the true or ideopathic affection. This last occurs chiefly in those who are above middle age, and is most common in men. It commences usually as a nodular thickening in the neighbourhood of the transverse line across the palm of the hand near the roots of the fingers. Professor Anderson has recently delivered some instructive lectures on this subject at the College of Surgeons. He considers that this disease is not due to friction, or to gouty deposits, but to a distinct micro-organism, which is introduced by scratches, especially by finger nails. Until this microbe has been found we must consider the pathology of the condition as *sub judice*. Whatever the cause may be, the bands of fibrous tissue which form do not belong to any of the fascias of the hand, but are new formations.

The plan of treatment adopted by Dupuytren was that of free division of the constricting bands. This caused large open wounds, and ill results sometimes followed. M. Jules

Guérin introduced the subcutaneous method, which has been so successfully carried out by Mr. Adams in this country. It must be noted that several operations are frequently necessary to effect a cure; and this only takes place if careful adjustment of splints is carried out for a long period.

The introduction of antiseptics has made the "open" operations practicable; and it is quite a question whether this is not in most cases the best, for the following reasons: 1. If the cuts be made subcutaneously, it is by no means easy to avoid injury to the tendon; (2) the nerves may be cut if there is much deviation from the middle line; (3) several operations, or at least several punctures, are necessary. Whereas, if the incision be made longitudinally in the middle line, and a dissection made, it is easier to avoid the above catastrophes, and there is little, if any, extra danger. If it is necessary to cut the skin transversely, a side flap may be made to fill the gap, as suggested by Professor Anderson; but the writer of this article has found that the gaping wounds so made heal over very quickly if the finger is carefully fastened to the splint and no subsequent contraction occurs.

2. *Contractions of the skin* occur as secondary consequences of any chronic form of finger deformity, and they follow burns and various other injuries. The operation required depends on the extent of surface involved, but the transplantation of flaps of skin is often necessary. These cases are not very satisfactory. By far the best treatment is the preventive one. They generally follow the neglect of the use of splints and passive movements.

Cases have been recorded of supposed primary contraction of the skin of the fingers, but these are probably all to be considered as secondary.

3. *Contraction of the tendons* occurs from central nervous disease causing spastic conditions of one set of muscles or paralysis of others. These deformities can only be partially relieved unless the original disease is cured, which is not, of course, usual.

Injury to a nerve or muscle, also, by paralysing movement in one direction, is a cause of contraction. Little can be done for this.

It may be mentioned here that rupture of the tendons, either from violent wrenches or from cuts, should be operated on as soon as possible. Delay is dangerous, not only because of the shrinking of the severed ends, but because of the new attachments they may form. In hunting for the proximal end of a cut tendon it must be remembered that any microbes introduced into the sheath may set up inflammation, which may for ever cripple the finger or even the whole hand. The forceps, &c., should, therefore, be scrupulously cleaned and sterilised. When the ends are found it is well to use strong catgut, and to put the sutures in as deeply as possible. With the greatest care the result is frequently *nil*. It is not, however, absolutely necessary that the two cut ends be brought into close contact, for these operations have occasionally succeeded in restoring motion to the finger even when there has been an unavoidable gap left. This, probably, is due to the formation of a blood clot in the sheath, which becomes organised and fastens the two ends together as "callus" fastens the separated ends of bone.

